# Macrophage Biology in Human Granulomatous Skin Inflammation

**DOI:** 10.3390/ijms24054624

**Published:** 2023-02-27

**Authors:** Henning Klapproth, Manuel Huerta Arana, Mario Fabri

**Affiliations:** 1 Department of Dermatology and Venereology, Faculty of Medicine, University of Cologne, and University Hospital of Cologne, 50937 Cologne, Germany; 2 Center for Molecular Medicine Cologne (CMMC), Faculty of Medicine, University of Cologne, 50937 Cologne, Germany

**Keywords:** granuloma, macrophage, metabolism, interferon-γ, immune function, granuloma annulare, sarcoidosis, leprosy

## Abstract

Cutaneous granulomatoses represent a heterogeneous group of diseases, which are defined by macrophage infiltration in the skin. Skin granuloma can be formed in the context of infectious and non-infectious conditions. Recent technological advances have deepened our understanding of the pathophysiology of granulomatous skin inflammation, and they provide novel insights into human tissue macrophage biology at the site of ongoing disease. Here, we discuss findings on macrophage immune function and metabolism derived from three prototypic cutaneous granulomatoses: granuloma annulare, sarcoidosis, and leprosy.

## 1. Introduction

Macrophage (Mᶲ) infiltration is the hallmark of cutaneous granulomatoses, a heterogeneous group of skin diseases, which exists with or without systemic manifestations. Granulomatous skin inflammation can be triggered by various pathogens (including bacteria, parasites, and fungi), foreign bodies, malignancies, and drug reactions, or it can occur as a seemingly causeless immunologic dysregulation (for example, in granuloma annulare (GA) or sarcoidosis) [1]. Scientifically, human cutaneous granulomatoses provide extraordinary model diseases for learning about human Mᶲ biology, providing an opportunity for investigating immunity, both sterile and infectious, at the site of disease activity.

Clinically, cutaneous granulomatoses typically show localized or disseminated erythematous, brownish to yellowish plaques, with or without epidermal involvement, or subcutaneous nodules. The precise function of granuloma remains unclear. It is generally accepted that the human body forms granuloma when the immune system identifies a potentially harmful agent that cannot be eliminated [2]. However, in the case of infectious diseases, granuloma formation may also constitute an immune evasion strategy by the pathogen [3]. Histologically, granulomatous responses can demonstrate distinct interstitial or nodular patterns with palisading, suppurative, sarcoid, or tuberculoid features; however, their defining feature is infiltration by Mᶲ [1]. Cutaneous granulomatoses are frequently misdiagnosed. Moreover, their treatment is challenging due to limited options, side effects, and highly variable responses. Largely, this is due to the incomplete understanding of the pathophysiology underlying cutaneous granulomatoses and, consequently, to unfitting pharmacological approaches. The heterogeneous, and often limited, responses of patients to therapy result in a strong need for studying the immunologic state of Mᶲ biology in infectious and non-infectious granulomatous skin diseases.

For a long time, cutaneous granulomatous inflammation has remained poorly understood at the molecular level. In recent years, however, technological advances have contributed to elucidating the molecular mechanisms involved in granulomatous skin inflammation, and we are beginning to gain a much better understanding of Mᶲ biology in these unique inflammatory reactions. The scientific advances, fueled by the novel research techniques, lay the groundwork for this review. We discuss novel insights into human Mᶲ biology derived from studies of three prototypical cutaneous granulomatoses, namely, granuloma annulare (GA), sarcoidosis, and leprosy. We point out shared and distinguishing characteristics of Mᶲ biology in different granulomatous inflammatory states of the skin. By doing so, the review provides scientific guidance regarding basic molecular concepts of the covered diseases and more general concepts of granulomatous inflammation.

## 2. Granuloma Annulare

GA, a prototype of necrobiotic granuloma [4], is a chronic inflammatory skin disorder whose etiology remains unknown since it was first described in 1895 [5]. Histologically, GA typically features palisading Mᶲ circumscribing paucicellular areas composed of altered collagen and mucin as well as peripheral T-cell infiltrates [6,7]. Although generally classified as a non-infectious disease, borrelia and viral infections have been suggested as triggering factors [8,9,10,11,12]. The lack of knowledge of the molecular mechanisms involved in the pathogenesis of GA limits the therapeutic options.

To improve therapeutic strategies in GA, current efforts focus on elucidating the cellular composition and molecular mechanisms driving the pathogenesis of GA. Despite being limited in number and partly displaying conflicting results, recent studies deepening our understanding of GA are consistent with a key role of IFN-γ-mediated Mᶲ activation [13,14,15]. Thus, these studies promote the concept of a Th1-type inflammatory reaction pattern. Using single-cell RNA sequencing (scRNAseq) in lesional skin from patients before and after treatment with the JAK/STAT inhibitor tofacitinib and healthy skin controls, Wang et al. identified and analyzed the transcriptomic profile of Mᶲ and other cells, including T cells in GA [15]. Data integration suggests that chronically activated Th1 cells recruit circulating monocytes into GA lesions via CCL3 and CCL4 [15]. Consequently, IFN-γ produced by Th1 cells polarizes these monocytes into activated Mᶲ [15]. These Mᶲ express a transcriptional signature that resembles an inflammatory M1-like Mᶲ pattern (*CXCL9, CXCL10, IFI27, FCGR1A*). Nevertheless, anti-inflammatory M2 markers (*FCGR1A* and *CD163*) were also upregulated [15]. Noteworthy, the common M1/M2 Mᶲ polarization paradigm may not represent the human Mᶲ spectrum in GA. Under in vivo conditions, human Mᶲ are subjected to a large number of stimuli, in a time- and space-dependent manner, which do not resemble pure LPS, IFN-γ, or IL-4 stimulus conditions of the classical in vitro models. Nutrient competition makes functional regulation of Mᶲ more complex [16,17,18]. In summary, Wang et al. proposed an attractive concept for GA, in which activated Mᶲ express SPP1 (osteoponin) and FN1 (fibronectin). These signaling molecules in conjunction with IFN-γ drive changes in fibroblast function and extracellular matrix composition. Their findings define a central role of IFN-γ and its influence on Mᶲ for disease initiation. The elevated expression of IFN-γ in GA pathophysiology was acknowledged two decades ago [14,19]. Early studies described a co-expression with TNF-α [14,20], and such findings have been substantiated by reports of several cases demonstrating efficacy of adalimumab and infliximab, two TNF-α inhibitors, in the treatment of GA [21,22]. Nevertheless, anti-TNF treatments have not shown consistent results, and paradoxical induction of GA by TNF-α has also been described [23,24]. Despite the collective evidence that IFN-γ is the signature cytokine, Min et al. reported massive upregulation of *IL-4* and *IL-31* by bulk qRT-PCR on GA skin lesions [13], contrasting with the results of Wang et al. [15].

To characterize the cellular composition and phenotype in normal human skin and in different inflammatory skin conditions, among them GA and leprosy, Hughes et al. developed and applied a new high-throughput scRNAseq technique called “Seq-Well S3” that uses second-strand synthesis to improve transcript capture and provide increased sensitivity in gene detection [25]. Their analyses of GA uncovered a diverse group of cytotoxic cells containing NK cells, γβ-T cells, and a cluster of immature cytotoxic T cells. Deeper analysis of CD8^+^ T cells detected a sub-group of activated CD8^+^ T cells expressing elevated amounts of several inflammatory cytokines (TNF, CCL4, and XCL1), which act to recruit and activate Mᶲ, specific affinity receptors (FASLG and TNFRSF9), and transcription factors (KLF9 and EGR2) in GA [25]. Distinct fibroblast populations displaying upregulation of *SPOCK1, CRLF1, COMP,* and the protease inhibitors 16 and *ITIH5*, which inhibit the function of MMP2 and help maintain dermal hyaluronic acid, respectively, were also reported in GA [25].

In the past decade, it has become clear that the crosstalk of metabolic pathways with immune signaling cascades drives Mᶲ phenotypic and functional states [17]. Consequently, in granulomatous inflammation, a process in which Mᶲ are the predominant cells, numerous studies show a direct relationship with immunometabolism [26,27,28,29]. As early as 1977, Umbert and Wilkenmann, prior to the rise of immunometabolism, reported large amounts of succinic dehydrogenases in the cellular infiltrate of patients with GA [7]. Interestingly, it is now clear that succinate dehydrogenase supports metabolic reprogramming to drive inflammatory Mᶲ [30]. IFN-γ is a strong inducer of indoleamine-2,3-dioxygenase (IDO), an enzyme that degrades the essential amino acid (AA) tryptophan in human Mᶲ [31,32]. Not surprisingly, IDO expression has been reported in GA Mᶲ [33], providing a possible mechanism by which IFN-γ regulates mTORC1 activation and metabolic reprogramming in Mᶲ. Note that Mᶲ concentrations of leucine, the most potent mTORC1 activating AA, do not decrease in response to IFN-γ-induced activation of human Mᶲ, whereas tryptophan does [31]. Therefore, IFN-γ may regulate mTORC1 activation in Mᶲ via tryptophan depletion given a partial dependency of mTORC1 on this AA for its activation [31].

Taken together, available evidence supports the concept that IFN-γ-driven Mᶲ immune-functional and metabolic programs are central to the pathophysiology of GA [13,14,15], which can also explain the increasingly reported successful treatment of GA with JAK/STAT inhibitors [34,35,36].

## 3. Cutaneous Sarcoidosis

The current understanding of sarcoidosis is that of an idiopathic, inflammatory disorder characterized by the aggregation of Mᶲ [1]. The pathological hallmark of sarcoidosis is the formation of non-caseating granuloma that may involve almost any organ of the body, including, notably, the skin [1]. In the yet incompletely understood process of granulomatous immune response, innate and adaptive immune cells as well as matrix-associated cells are recruited to the tissue by various chemokines, cytokines, and other signaling molecules [1]. Conceptually, the pro-inflammatory molecular pattern promotes aggregation, cell hypertrophy, and possibly fusion of myeloid monocytic cells leading to granuloma formation and upholding local inflammation [37]. This process seems mechanistically comparable to a local immune pathology and is thought to be (co-)initiated by T cells that recruit monocytes from the blood to the tissue [38]. At least in the case of pulmonary sarcoidosis, previously obtained data suggest that Th1, Th2, and Th17 T-cell phenotypes play a pathomechanistic role, involving their potential to secrete characteristic cytokines driving Mᶲ polarization, such as IFN-γ, TNF-α, and IL-17 [39,40]. In general, the clinical presentation of sarcoidosis displays association with specific HLA antigens [41,42], which emphasizes the influence of the adaptive immune system on this primarily Mᶲ disease. Among the inciting stimuli resulting in granuloma formation in sarcoidosis, different environmental, microbial, and (epi-)genetic factors have been the subject of discussion. However, in-depth understanding on the pathophysiology of cutaneous sarcoidosis is missing. This is, at least in part, due to varying concepts of granuloma initiation and formation that have originated from different animal models used for investigating sarcoidosis in the past. Either these studies have not been sufficient to provide molecular core mechanisms for cutaneous sarcoidosis or models have not been adequately applicable to human Mᶲ biology [43,44].

Recently, the central role of Mᶲ in driving the pathophysiology of cutaneous sarcoidosis has been reemphasized in murine and human models [26,45,46,47]. Investigations of human data sets of lung tissue identify Mᶲ as the key drivers of granuloma formation but also granuloma maintenance and even resolution, thereby supporting the concept of a continuous spectrum of Mᶲ biology [26,48,49]. Studying human skin sarcoidosis, Damsky and colleagues pointed out the implication of type I cytokines, IFN-γ in particular, in activating human sarcoid Mᶲ [45]. Consistently, earlier results derived from microarray gene data sets of cutaneous sarcoidosis showed an upregulated IFN-γ pathway [50]. The implication of IFN-γ on Mᶲ in human skin is underlined by its effects on Mᶲ immunologic reprofiling: in cutaneous sarcoidosis, IFN-γ-related expression of downstream translational targets includes *STAT1, CEBPB, FN1, TREM1, CXCL9, CXCL10, IL-6*, and others [45]. In this manner, T-cell activation and stimulation of fibroblasts as well as auto-activation seem to be driven by Mᶲ.

IFN-γ appears to be related to disease activity of cutaneous sarcoidosis at different sites [45]. In the study of Damsky et al., immunologic gene expression patterns in the affected skin were markedly similar to those in pulmonary sarcoidosis samples [45]. Th2 and Th17 marker expression in both cutaneous and pulmonary samples of sarcoidosis patients was negligible, which contrasts findings in pulmonary sarcoidosis by other authors [45]. The role of IFN-γ in cutaneous sarcoidosis is reinforced by the observation that IFN-γ-inducible chemokines CXCL9 and CXCL10 are elevated in serum and tissue of patients with systemic sarcoidosis, and blood transcriptional profiles show IFN-γ-related signaling pathways in such patients [51,52]. These chemokines and other IFN-γ-related downstream translational targets were not only expressed by M1-like Mᶲ but also stromal cells, such as fibroblasts and endothelial cells [52,53]. Thus, IFN-γ has a pivotal role as the pro-inflammatory stimulus in granuloma formation-associated cells and as the key chemokine in upholding the T-cell recruitment and thereby amplifying the immune response [52,53]. While mechanisms of IFN-γ-induced JAK1/JAK2-STAT1 signaling have been extensively reviewed elsewhere [54,55], JAK inhibition has been successfully used in treating cutaneous sarcoidosis [27,35,56].

IFN-γ production by Th1 cells is linked to Mᶲ metabolic reprogramming in cutaneous sarcoidosis. IFN-γ-upregulated JAK signaling results in enhanced expression of STAT1 and its target genes [54,57]. Through this signaling axis, IFN-γ seems to influence human Mᶲ polarization and metabolism by promoting glycolytic enzymes and impairing mitochondrial OXPHOS, albeit almost all studies on Mᶲ metabolism have investigated IFN-γ only in combination with LPS treatment [58,59]. Interestingly, gene set enrichment analyses of the sequencing data set from a patient with skin sarcoidosis treated with tofacitinib identified mTORC1 signaling and glycolysis, but also OXPHOS and fatty acid metabolism, next to “IFN-γ response” and “TNF-α signaling via NFκB” as inflammatory hallmarks of the affected patient skin [27,45]. Therefore, it stands to reason that metabolic changes in Mᶲ biology are the immunologic hub in cutaneous sarcoidosis, as most Mᶲ immune signals are somehow linked to metabolic changes in these cells.

One concept by which IFN-γ may drive Mᶲ metabolism is via interplay with HIF1α and mTOR signaling. In human monocyte-derived macrophages (MDM), IFN-γ-mediated metabolic changes are regulated by mTOR and MAPK/PI3K-Akt signaling [31,60]. In a murine model of sarcoidosis, mTOR activation leads to spontaneous generation of skin granulomas, and in pulmonary sarcoidosis in humans, activated mTOR signaling is associated with progressive disease [26]. Additionally, mTOR-mediated metabolic changes may encompass enhanced glycolysis [26], which is supported by the clinical evidence that sarcoidal granulomas in patients with systemic sarcoidosis express a high uptake capacity for glucose on FDG-dependent imaging [61].

Su et al. found that in primary human monocytes and MDM, IFN-γ reprograms metabolism to alter inflammatory responses induced by TLR ligands [31]. IFN-γ primarily reprograms monocyte metabolism by exerting selective effects on the translation efficiency of distinct mRNAs via targeting mTORC1 and MNK kinases that converge on the selective regulator of translation initiation eIF4E [31]. Interestingly, these effects of IFN-γ on Akt-mTORC1 and MNK-eIF4E signaling appear to be cell context dependent and increase with the duration of IFN-γ exposure [31]. Thus, future studies in granuloma models at different time points in parallel with further consideration of the role of immunometabolism in Mᶲ may provide additional insights into the pathophysiology of granuloma-tous, chronic processes in the skin.

HIF1α is known as a central gate keeper in Mᶲ metabolism and is predominantly expressed in pulmonary granulomas in sarcoidosis, where HIF1α increases Glut1 expression and levels of IL-1β in alveolar Mᶲ and monocytes [62,63]. At the same time, the metabolite succinate regulates cytokine expression and various other, mostly pro-inflammatory, signals, at least in mouse macrophages, through HIF1α [64]. However, there is conflicting data on mTOR regulated metabolism in human Mᶲ originating from different models and resulting in limited comparability to cutaneous sarcoidosis [26,65,66,67,68,69]. Nevertheless, these studies point to a fundamental metabolic rewiring of human Mᶲ in skin sarcoidosis, including changes in core energy pathways, such as glycolysis, OXPHOS, FAO, and lipid biosynthesis. Therefore, investigating metabolic pathways in cutaneous sarcoidosis will further unravel mechanisms that are initiating and upholding disease activity in the skin and may arise as promising therapeutic targets.

## 4. Leprosy

Leprosy, which is caused by the intracellular pathogen *Mycobacterium* (*M.) leprae*, can manifest as a chronic granulomatous disease predominantly affecting the dermal compartment of the skin and peripheral nerves. In leprosy, granuloma formation seems to constitute a defense mechanism combating microbial pathogens [70]. Leprosy has served as an important model for studying the dynamics of innate immune responses to (mycobacterial) infection in the past decades. Clinically, the presentation of leprosy is notedly variable, and, therefore, the disease is clinically considered as a bi- to multipolar spectrum. Tuberculoid leprosy (T-lep)—a paucibacillary, immunological well-controlled form of the disease with organized granuloma—represents one end of the spectrum [71]. In contrast, lepromatous leprosy (L-lep), lying at the other end of the spectrum, is a multibacillary, immunological poorly controlled form showing disorganized granulomatous inflammation. In between the two poles and depending on the clinical classification, dimorphous or borderline forms of leprosy (BL) exist, yet these have rarely been a starting point of systematic investigations.

In this multi-faceted disease, clinical presentation critically depends on local immunologic interplay between the adaptive and the innate immune system and its antimicrobial potential [72,73]. Unsurprisingly, granuloma composition, spatial distribution of involved cell types, Mᶲ morphology, and lymphocytic infiltrates differ distinctly across leprosy manifestations [71]. The nature and quality of the innate immune response is fundamentally linked to these clinically different manifestations. This phenomenon is well reflected by a continuous immunologic spectrum of Mᶲ polarization states in the different clinical presentations, related to respective specific profiles, extending beyond the simplifying M1/M2 paradigm. Precise Mᶲ-inherited causative cues regarding the fate of the organism after intracellular infection are missing, although various susceptibility factors of Mᶲ, such as genetic *NOD2* and *TLR1* polymorphisms, have been described [74,75].

Distinctions between transcriptional programs in human skin samples from L-lep and T-lep have provided great insights into Mᶲ biology in human intracellular infections. Generally, L-lep lesions exhibit *M. leprae*-loaden Mᶲ with high phagocytic capacity of mycobacteria and of oxidized low-density lipoproteins, accumulating intracellularly, which promotes the characteristic L-lep Mᶲ foamy appearance [76,77]. L-lep Mᶲ, however, fail in providing effective antimicrobial activity [76,77]. This reduction of antimicrobial capacities in L-lep is, for instance, linked to the inhibition of autophagy by *M. leprae* itself [78]. Interestingly, single-cell sequencing data of human adipose tissue Mᶲ show similar translational profiles to Mᶲ of L-lep lesions in terms of polarization states and cell metabolic hints that promote “lipid metabolism” in TREM2 Mᶲ in L-lep [70,79].

A major cytokine pattern responsible for instructing Mᶲ in L-lep is the type I interferon (IFN-α/β) and its downstream targets, such as IL-10 [80,81]. These cytokines regulate iron uptake, possibly by CD163-mediated endocytosis of haptoglobin-hemoglobin and thereby facilitating mycobacterial survival, they enhance lipid phagocytosis mediated by scavenger receptors and suppress antimicrobial defense mechanisms, e.g., by attenuating production of antimicrobial peptides and autophagy. In addition, they might engage in cell metabolism by inducing *Arg1* expression [77,81,82,83,84,85,86]. However, the metabolic program of Mᶲ in L-lep remains far from revealed. At the same time, unraveling the Mᶲ immune metabolism in L-lep might provide valuable insights into the causes of ineffective antimicrobial defense.

Opposed to the role of type I interferons in L-lep that accompany a predominantly Th2 type immune response, IFN-γ has been identified as an important regulator in other forms. IFN-γ is linked to immune control in T-lep [80,87]. Mᶲ become prominently enriched in T-lep lesions forming granulomas [76]. Moreover, IFN-γ is the driving cytokine in reversal reaction (RR), a process by which multibacillary disorganized L-lep Mᶲ arrangements, spontaneously or with the help of chemotherapy, transition to paucibacillary T-lep granuloma. This process is associated with a polarization shift towards an inflammatory M1-like phenotype and increasing Mᶲ antimicrobial capacities [70,76,78,88]. IFN-γ has been identified as an upstream regulator of a vitamin D-dependent antimicrobial pathway in Mᶲ of T-lep and RR, as also described in other models [70,77,89,90]. This is in line with the general notion of IFN-γ-activated, inflammatory M1-like Mᶲ responsible for host defense. The Mᶲ antimicrobial capacities are linked to the adequate maturation of the autophagosome-by-autophagosome cargo receptors, such as SQSTM1/p62 or NBR1, that deliver intracellular pathogens to the lysosome for degradation [78]. Expression of autophagy-associated genes is higher in T-lep than in L-lep specimens [78]. In parallel, cathelicidin (*CAMP*) and β-defensin 2 (*DEFB4A*), which contribute to lysosomal killing of the pathogen, show higher mRNA expression in T-lep [78]. In contrast to these autophagolysosomal processes, Inkeles et al., using computational deconvolution analysis, reported enhanced “endosomal” and “phagocytotic” pathways as well as augmented “lipid binding” of Mᶲ in L-lep lesions (see above) [76]. Focusing on the role of interferons in this scenario, it is important to point out that MMP-12 (known to be expressed by Mᶲ, i.e., and induced by pro-inflammatory molecular patterns in DC in vitro) may be capable of directing the immunologic pattern in leprosy by blocking type I interferons [76,91,92]. In the notion of a Mᶲ biological switch, IFN-γ-dependent activation of vascular endothelial cells facilitates M1-like Mᶲ polarization of monocytes [93]. Directional IFN-γ-dependent reprogramming of Mᶲ during RR is also associated with metabolic changes in Mᶲ biology. During RR, transcriptional profiles of whole blood analyses report “mitochondrial electron transport” and “OXPHOS” as strongly enriched pathways [88].

The study by Hughes et al., using “Seq-Well S3” (see above), observed a cell population with enriched expression of Th-17 canonical markers in half of the analyzed samples [25]. *ROR-γt*, a Th17-defining transcription factor that may be associated with Mᶲ recruitment in chronic inflammation and granuloma formation [94], was among the enriched genes in this T-cell population [25]. Enrichment of genes related to IFN-γ responses was observed in Langerhans cells [25]. Additionally, Hughes et al. also detected a unique Mᶲ population in a skin sample of one leprosy patient defined by expression of extracellular proteases (LYZ, CHIT1, and CHI3L1) [25]. Interestingly, CHIT1 was reported to be significantly upregulated in serum from sarcoidosis patients, and its pharmacological inhibition demonstrated efficacy as a therapeutic approach in murine models of granulomatous inflammation [95].

Recently, Ma et al. provided an insightful view on the underlying pathophysiology of human leprosy in L-lep and its RR by applying single-cell and spatial sequencing to skin specimens [70]. Strikingly, M1-like Mᶲ induced by IFN-γ of Th17 cells in T-lep or Mᶲ that have undergone transition during RR under immunologic guidance by IFN-γ spatially aggregate in the core of T-lep granuloma in close microanatomical distance to T cells [70]. In line with the immunologic changes following enhanced IFN-γ signaling, IL-1β derived from dendritic cells and Langerhans cells in RR is assumed to exert synergistic effects with IFN-γ on numerous antimicrobial genes [70]. While Mᶲ certainly are the key players in host defense against *M. leprae*, other major cell entities, including keratinocytes and fibroblasts, are reported to express antimicrobial gene sets as well [70]. Interestingly, antimicrobial peptides produced by keratinocytes and fibroblasts are able to enhance the antimicrobial capacities of Mᶲ against *M. leprae,* resulting in decreased *M. leprae* viability, as shown in MDMs [70]. Furthermore, the findings of Ma et al. add proof to the more commonly accepted notion that polarization states of Mᶲ are plastic [70]. In the case of leprosy, this means that TREM2 Mᶲ in L-lep have the capability of transitioning into M1-like Mᶲ being the predominant Mᶲ type in RR and T-lep, a process that is mainly driven by IFN-γ [70].

Taken together, collective evidence strongly supports the concept that IFN-γ instruction of the Mᶲ response critically orchestrates the course of *M. leprae* infection and, moreover, granuloma composition and spatial distribution of involved cell types.

## 5. Conclusions and Outlook

Mᶲ biology in human granulomatous skin diseases is gaining increasing interest in the scientific community. Here, we reviewed three representative granulomatous skin diseases of palisading, sarcoidal, and tuberculoid histology, respectively. The purpose of this review was to highlight leading immunologic patterns of Mᶲ biology in the covered granulomatous skin diseases and to compile disease-specific immunologic aspects of Mᶲ biology. In all reviewed disease entities, granuloma formation presumably resulted from monocyte recruitment into the skin [15,45,70]. Furthermore, IFN-γ-dependent activation of Mᶲ steers their polarization pattern and function in granuloma pathophysiology, and IFN-γ is involved in the initiation of cutaneous granuloma formation. In line with this notion, it is unsurprising that IFN-γ treatment in other clinical settings is able to induce granulomatous skin eruptions [96,97]. Conversely, clinical improvements in granuloma annulare as well as sarcoidosis have most profoundly been achieved by inhibition of IFN-γ-downstream signaling via JAK/STAT pathways using specific inhibitors, such as tofacitinib [15,27,35,56]. It should be noted that, although only three different representative cutaneous granulomatous inflammatory entities are discussed in the present review, this group of diseases comprises many more (Table 1). It seems reasonable to speculate that there are distinctive but also shared molecular mechanisms involved in the pathophysiology of these different cutaneous granulomatoses. A role for IFN-γ-induced programming of inflammatory Mᶲ was also recently described in non-granulomatous inflammation, specifically psoriasis [98].

Across models, diseases, and disciplines, a great scientific interest concerning metabolism of different Mᶲ polarization states has rapidly developed [31,99,100,101]. However, there are only a few studies integrating the knowledge of Mᶲ immunometabolism into the field of human inflammatory diseases. Collective evidence suggests that IFN-γ is a pivotal regulator of Mᶲ metabolic reprogramming. By applying gene expression profiling of IFN-γ-treated human Mᶲ, our group previously identified a host defense network [102] associated with key metabolic pathways (unpublished data).

According to the simplified M1/M2 dogma, proinflammatory M1-like Mᶲ, normally stimulated by LPS and IFN-γ, have a metabolic shift towards aerobic glycolysis along with OXPHOS suppression [17,59,103]. However, considering that lineage diversification and plasticity of Mᶲ are key aspects of their functionality [16,104,105], defining glycolysis as proinflammatory may be oversimplifying. Strikingly, Su et al. showed, by pathway analysis in IFN-γ-treated human Mᶲ, that metabolism-related genes with enhanced expression were significantly associated with OXPHOS and mitochondrial pathways [31]. These identified pathways exhibit similarities to the IFN-γ-driven macrophage program observed in skin GA, sarcoidosis, and leprosy as mentioned above [15,27,88]. Of note, metabolic pathways in Mᶲ were among the most altered in leprosy [70,88].

In summary, the pathophysiology of granulomatous skin diseases provides an excellent field for further research on principles of human Mᶲ metabolism and interplay with immune signaling cascades, e.g., within the IFN-γ signaling axis. Advances derived from a better understanding of the inflammatory process at a molecular level, as summarized here, will result in better treatment options. Several current concepts center on relatively broad-acting biological disease-modifying drugs, i.e., JAK inhibitors. However, as the state of the art continues to evolve, high-definition refinement of inflammatory patterns might allow for even more target-driven treatments.

## Figures and Tables

**Table 1:** Examples of granulomatous skin diseases. Adapted from (1).

Non-Infectious	Infectious
Sarcoidosis Lupus erythematosus Rheumatoid arthritis (interstitial granulomatous dermatitis) Inflammatory bowel disease Foreign bodies Gout nodules Granuloma annulare Necrobiosis lipoidica Necrobiotic xanthogranuloma Rosacea Acne inversa Orofacial granulomatosis Granulomatous mycosis fungoides Granulomatous drug reactions	Tuberculosis Non-tuberculous (atypical) mycobacterioses Leprosy Syphilis Toxoplasmosis Leishmaniasis Histoplasmosis Cryptococcosis Brucellosis

## Data Availability

No new scientific data was created for this review.

## Abbreviations

AA	Amino acid
Akt	Serine/threonine kinase
BLCAMP	Borderline leprosyCathelicidin antimicrobial peptide
CCL3	Chemokine (C-C motif) ligand 3
CCL4	Chemokine (C-C motif) ligand 4
CD163	Cluster of differentiation 163
CHIT1	Chitinase 1
CHI3L1	Chitinase-3-like protein 1
COMP	Cartilage oligomeric matrix protein
CRLF1	Cytokine receptor-like factor 1
CXCL9	Monokine induced by gamma interferon
CXCL10	10 kDa interferon gamma-induced protein
DEFB4A	Beta-defensin 2
EGR2	Early growth response protein 2
eIF4E	Eukaryotic initiation factor 4E
FAO	Fatty acid oxidation
FASLG	Fas ligand (CD95L or CD178)
FCGR1A	Fc gamma receptor Ia (CD64)
FDG	Fluorodeoxyglucose
FN1	Fibronectin
GA	Granuloma annulare
Glut1	Glucose transporter 1
HIF1α	Hypoxia-inducible factor 1-α
IDO	Indoleamine 2,3-dioxygenase
IFI27	Interferon alpha-inducible protein 27
IFN-γ	Interferon-γ
IL-	Interleukin-
ITIH5	Inter-alpha-trypsin inhibitor heavy chain 5
JAK	Janus kinase
KLF9	Kruppel like factor 9
LPS	Lipopolysaccharide
L-lep	Lepromatous leprosy
LYZ	Lysozyme
MAPK	Mitogen-activated protein kinase
MDM	Monocyte-derived macrophage
MNK	MAPK-interacting kinase
MMP	Matrix metalloproteinase
mTORC1	Mammalian target of rapamycin
Mᶲ	Macrophage
M. leprae	Mycobacterium leprae
NFκB	Nuclear factor κ-light-chain-enhancer of activated B cells
NK	Natural killer
NOD	Nucleotide-binding oligomerization domain-containing protein
OXPHOS	Oxidative phosphorylation
PI3K	Phosphoinositide 3-kinase
qRT-PCR	Quantitative reverse transcription polymerase chain reaction
RR	Reversal reaction
ROR-γt	RAR-related orphan receptor gamma transcription factor
Th1	T helper 1
T-lep	Tuberculoid leprosy
scRNAseq	Single-cell RNA sequencing
SPOCK1	Testican-1 gene
SPP1	Osteoponin
SQSTM1/p62	Sequestosome-1/ubiquitin-binding protein p62
STAT1	Signal transducer and activator of transcription 1
Th	T helper
TLR	Toll-like receptor
TNF-	Tumor necrosis factor
TNFRSF9	Tumor necrosis factor receptor superfamily member 9 (CD137)
TREM	Triggering receptor expressed on myeloid cells
